# The Distribution of Ocular *Chlamydia* Prevalence across Tanzanian Communities Where Trachoma Is Declining

**DOI:** 10.1371/journal.pntd.0003682

**Published:** 2015-03-27

**Authors:** Salman A. Rahman, Sheila K. West, Harran Mkocha, Beatriz Munoz, Travis C. Porco, Jeremy D. Keenan, Thomas M. Lietman

**Affiliations:** 1 F.I. Proctor Foundation, San Francisco, California, United States of America; 2 Dana Center for Preventive Ophthalmology, Johns Hopkins University, Baltimore, Maryland, United States of America; 3 Department of Ophthalmology, University of California, San Francisco, San Francisco, California, United States of America; 4 Department of Epidemiology & Biostatistics, University of California, San Francisco, San Francisco, California, United States of America; University of California San Diego School of Medicine, UNITED STATES

## Abstract

**Background:**

Mathematical models predict an exponential distribution of infection prevalence across communities where a disease is disappearing. Trachoma control programs offer an opportunity to test this hypothesis, as the World Health Organization has targeted trachoma for elimination as a public health concern by the year 2020. Local programs may benefit if a single survey could reveal whether infection was headed towards elimination. Using data from a previously-published 2009 survey, we test the hypothesis that *Chlamydia trachomatis* prevalence across 75 Tanzanian communities where trachoma had been documented to be disappearing is exponentially distributed.

**Methods/Findings:**

We fit multiple continuous distributions to the Tanzanian data and found the exponential gave the best approximation. Model selection by Akaike Information Criteria (AIC_c_) suggested the exponential distribution had the most parsimonious fit to the data. Those distributions which do not include the exponential as a special or limiting case had much lower likelihoods of fitting the observed data. 95% confidence intervals for shape parameter estimates of those distributions which do include the exponential as a special or limiting case were consistent with the exponential. Lastly, goodness-of-fit testing was unable to reject the hypothesis that the prevalence data came from an exponential distribution.

**Conclusions:**

Models correctly predict that infection prevalence across communities where a disease is disappearing is best described by an exponential distribution. In Tanzanian communities where local control efforts had reduced the clinical signs of trachoma by 80% over 10 years, an exponential distribution gave the best fit to prevalence data. An exponential distribution has a relatively heavy tail, thus occasional high-prevalence communities are to be expected even when infection is disappearing. A single cross-sectional survey may be able to reveal whether elimination efforts are on-track.

## Author Summary

Trachoma is the leading infectious cause of blindness and the World Health Organization plans to eliminate it as a public health concern worldwide by the year 2020. It can be difficult for local trachoma programs to assess whether disease is headed towards elimination in their area. Mathematical infectious disease models describe that when a disease disappears, its prevalence across communities in that area form an exponential distribution. However, this theorem has never been tested with field data. In this study, we take trachoma prevalence data from Tanzania, in an area where trachoma was known to be disappearing, and find that the prevalence forms an exponential distribution. The implications of this study could be applied to other infectious diseases to provide evidence that prevalence is headed towards elimination.

## Introduction

Epidemic models hypothesize that the prevalence of infection across communities where an infectious disease is disappearing should approach an exponential distribution. Simulations of mass treatments and decreasing transmission support this.[1–3] However, these epidemic models typically assume similar transmission parameters across communities, while observational studies suggest transmission heterogeneity even amongst neighboring communities.[4] If this hypothesis is consistent with field data, public health stakeholders would benefit by having the ability to forecast prevalence and learn whether a disease was on its way to elimination.

Trachoma programs offer an opportunity to test these models. Repeated ocular infection with *Chlamydia trachomatis* can result in irreversible blindness. Trachoma has been targeted by The World Health Organization (WHO) for elimination as a public health concern by the year 2020. Efforts rely on a multifaceted approach of mass antibiotic distributions to clear infection and hygiene improvements such as promoting facial cleanliness and latrine construction to reduce transmission. Whether due to intervention or secular trend, trachoma is clearly disappearing from many areas. [5–8]

A recent study suggested that the prevalence of infection across 24 communities in two separate regions of Ethiopia approached a geometric distribution, the discrete analog of the exponential. Longitudinal evidence confirmed trachoma was indeed disappearing in each of these two areas. [9] Here, we examine a far larger data set from a recent cross-sectional survey in Tanzania to determine the distribution of infection across communities that have received multiple rounds of mass antibiotics and where the prevalence of clinical signs of trachoma was known to be decreasing. We test the hypothesis that the distribution of Tanzanian prevalence data is exponential.

## Methods

In 1999, Tanzania implemented a trachoma control program within endemic districts through the National Trachoma Taskforce. Control efforts relied on mass azithromycin distribution to communities. During 2007–2008, 75 communities in 8 districts in Tanzania were randomly selected for a cross-sectional, population-based survey of infection, assessed by conjunctival swab and PCR for chlamydial DNA.[10] These communities had received at least three rounds of yearly azithromycin, with most having received 4–7 annual treatments. Pre-school children aged 5 years and under were surveyed as this age group is the reservoir of ocular chlamydial infection.[11,12] In 1999, mean prevalence of the clinical signs of trachoma (trachomatous inflammation—follicular or intense) in the 75 communities was 50% (ranged 17–79%). In the 2007–2008 cross-sectional survey, mean prevalence of clinical signs was 9.5% (ranged 0–28%). This latter study also found the mean PCR-determined *C*. *trachomatis* infection prevalence was 5.3% (ranged 0–25%). [10,13]

We assessed the fit of several continuous distributions to the 75 prevalence estimates from the 2007–2008 survey. It was assumed each community had a true, unobserved prevalence of infection and that the reported prevalence for each community was a sample from a binomial distribution given that true prevalence. We obtained parameter values by maximum likelihood estimation for the one-parameter (exponential, chi, chi-squared), two-parameter (beta, gamma, Weibull, normal, Cauchy, log-normal) and three-parameter (mixture exponential, generalized gamma) distributions, truncated between 0 and 1. The beta, gamma, Weibull, and generalized gamma include the exponential as a special case when the shape parameter is 1. The truncated normal and Gumbel distributions include the exponential as a limiting case as the location parameter approaches negative infinity. The mixture exponential includes the exponential as a special case when both rate parameters are equal or the proportion parameter is 1. The Cauchy, log-normal, chi, and chi-squared distributions do not include the exponential as a special case and were tested in this analysis to compare fit against the exponential.[14]

Models were ranked by sample size-corrected Akaike Information Criteria (AIC_c_) which penalizes a distribution for each additional parameter.[15] Bootstrap 95% confidence intervals for parameter estimates were determined by resampling communities (*n* = 999). We performed goodness-of-fit testing using the Cramer-von-Mises statistic to determine how unusual the observed data would be had they indeed come from an exponential distribution. To investigate spatial correlation of prevalence data, we used a Moran’s I statistic on communities in the Kongwa district. We performed two separate analyses: one using a weight matrix of inverse pair-wise distance between communities, and another using a binary weight matrix where a 1 signified neighboring communities and 0 signified non-neighbors. Neighbors were defined by those within the minimum distance needed such that each community had at least one neighbor. Statistical significance was determined by permutation test.

Lastly, we performed a sensitivity analysis by excluding villages in the Iramba district. The Iramba district contains four villages, all of which had 0 prevalence of infection and 0 prevalence of clinical signs of trachoma. The sensitivity analysis was performed by fitting the above-mentioned distributions to the restricted data, determining parameter values, and ranking by AIC_c_. All calculations were performed in *Mathematica* 9.0 (Wolfram Research, Champaign, Illinois).

### Ethics Statement

The study was carried out in accordance with the Declaration of Helsinki. Verbal consent was obtained from the local chiefs of each community before randomization. Verbal informed consent from each child participant’s guardian was obtained prior to the examination. This consent process was appropriate given the high rates of illiteracy in the study area and was approved by all institutional review boards.

## Results

**Fig 1:**
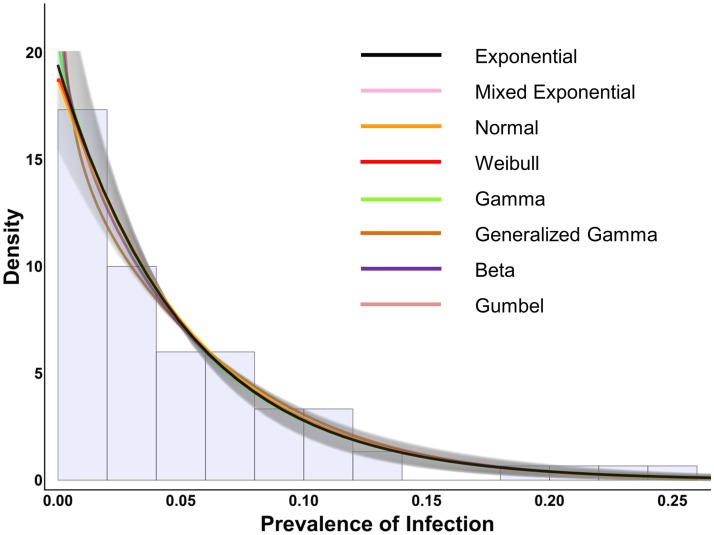
Exponential distribution and other distributions which mimic the exponential, fit to Tanzanian data. This figure shows the Tanzanian trachoma prevalence data as a histogram in the background along with the fits of various distributions which can mimic the exponential. The black line indicates the exponential distribution fit to the data, along with the 95% confidence interval as gray shading. All the other distributions give their best fit to the data when taking on parameter values that are consistent with the exponential, as shown by their fit within the 95% confidence interval (gray shading) of the exponential curve.

The exponential distribution had the lowest (best) AIC_c_. Note those distributions which include the exponential as a special or limiting case will always achieve a likelihood of having observed the data at least as high as the exponential. However, while the beta, Gumbel, normal, gamma, Weibull, generalized gamma distributions all had slightly better log likelihoods (slightly better fits), these distributions all contained additional parameters and therefore had higher (worse) AIC_c_ results. The sensitivity analysis yielded the same results as the main analysis, i.e. removing the 0 prevalence villages in the Iramba district had no effect and the exponential distribution gave the most parsimonious fit by AIC_c_. Results from the main analysis are summarized in Table 1. The fit of the exponential distribution to the data is shown in Fig. 1 along with the fit of those distributions which include the exponential as a special or limiting case.

**Table 1:** Fit of distributions, ranked by corrected Akaike Information Criteria (AIC_c_).

Truncated Distribution*	Log Likelihood	AIC_c_
**Exponential**	-211.791	425.637
***Distributions which include the exponential as a special or limiting case***		
**Beta**	-211.673	427.513
**Gumbel**	-211.745	427.656
**Normal**	-211.748	427.663
**Gamma**	-211.787	427.741
**Weibull**	-211.790	427.746
**Generalized Gamma**	-211.554	429.446
**Mixed Exponential**	-211.791	429.920
***Other distributions***		
**Cauchy**	-215.504	435.174
**Log-Normal**	-241.203	486.574
**Chi-Squared**	-246.164	494.382
**Chi**	-247.538	497.132

*All distributions were truncated between a prevalence of 0 and 1

**Fig 2:**
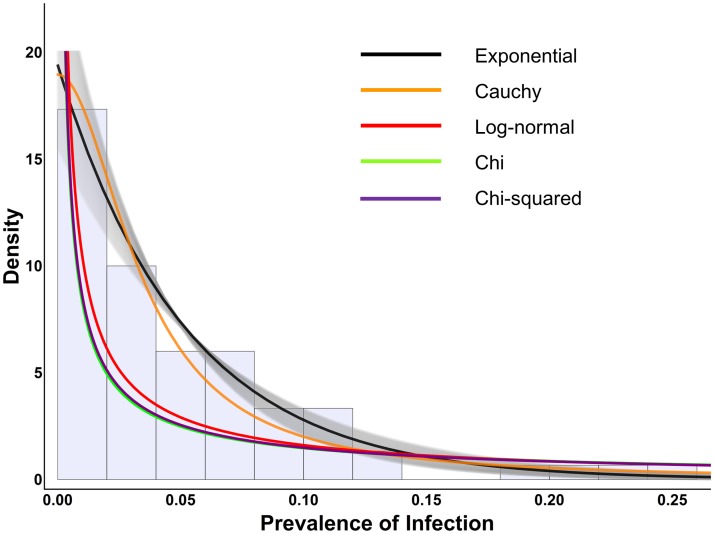
Exponential distribution and other distributions which cannot mimic the exponential, fit to Tanzanian data. This figure shows the Tanzanian trachoma prevalence data as a histogram in the background along with the fits of various distributions which cannot mimic the exponential. The black line indicates the exponential distribution fit to the data, along with the 95% confidence interval as gray shading. The best fit of all non-exponential distributions fall outside the 95% confidence interval (gray shading) of the exponential and give worse fit to the data.

The Cauchy, log-normal, chi, and chi-squared distributions do not include the exponential as a special or limiting case. These distributions gave far worse log likelihoods and AIC_c_ than the exponential. The fit of these distributions to the data is shown alongside the exponential in Fig. 2.

95% confidence intervals of the shape parameter estimates for the beta, gamma, Weibull, and generalized gamma distributions included 1, consistent with the special case of an exponential distribution (Table 2). The confidence interval for the location parameter of the truncated normal and Gumbel distributions included negative values, which again is consistent with the exponential. The mixture exponential distribution trivially reduced to a single exponential distribution as the proportion parameter estimate was 0.99 and the confidence interval included 1. With goodness-of-fit testing, we were unable to reject the hypothesis that the observed data came from an exponential distribution (*p* = 0.30). We found no evidence of spatial autocorrelation. Moran’s I using an inverse weight matrix was-.09 (*p* = 0.34) and Moran’s I using binary weight matrix was -0.02 (*p* = 0.85).

**Table 2:** Distribution parameter estimates with 95% confidence intervals.

Truncated Distribution	Shape Parameter	Parameter 2	Parameter 3
**Exponential**	**19.4** (15.48, 24.99)	**n/a**	**n/a**
***Distributions with exponential as a special or limiting case***			
**Beta**	**0.93** (0.64, 1.47)	**17.06** (11.39, 29.41)	**n/a**
**Gumbel**	**-3.75** (-6.87, -0.75)**	**1.20** (0.42, 1.98)	**n/a**
**Normal**	**-1.32** (-32.88, 0.01)**	**0.27** (0.06, 1.30)	**n/a**
**Gamma**	**0.98** (0.55, 1.65)	**0.05** (0.03, 0.08)	**n/a**
**Weibull**	**1.01** (0.81, 1.29)	**0.05** (0.04, 0.07)	**n/a**
**Generalized Gamma**	**0.54** (0.13, 1.39)	**0.09** (0.03,0.22)	**1.45** (0.89, 3.90)***
**Mixed Exponential**	**0.99** (0.15, 1.00)*	**19.41** (15.80, 625.17)	**16.76** (13.04, 21.17)
***Other Distributions***			
**Cauchy**	**0.02** (-0.01, 0.04)	**0.03** (0.02, 0.04)	**n/a**
**Log-Normal**	**0.00** (0.00, 0.05)	**4.31** (3.74, 4.90)	**n/a**
**Chi-Squared**	**0.52** (0.47, 0.62)	**n/a**	**n/a**
**Chi**	**0.25** (0.23, 0.30)	**n/a**	**n/a**

*Shape parameter for the truncated mixed exponential refers to the proportion parameter

**Shape parameter for the truncated normal and Gumbel distributions refers to the location parameter

***Parameter 3 for the generalized gamma refers to the second shape parameter

## Discussion

Here we show that chlamydial prevalence data from Tanzania are consistent with an exponential distribution. A dedicated control program had reduced the prevalence of clinical signs of trachoma 5-fold over 10 years in these Tanzanian communities. Of all distributions tested, the exponential had the most parsimonious fit to the data. Furthermore, the 95% confidence interval for the shape parameter estimate of each of the multi-parameter distributions included the special or limiting case of the exponential. Lastly, goodness-of-fit testing was unable to reject the hypothesis that the observed prevalence data came from an exponential distribution.

The Suceptible-Infected-Suceptible (SIS) epidemic model is used to study the transmission dynamics of pathogens, such as *C*. *trachomatis*, which can repeatedly infect individuals. In its simplest form, this model divides the population into two compartments: those who are susceptible to a disease and those who are infected. Members of the population flow between compartments at rates that reflect how transmissible the disease is and how quickly one recovers from infection. The model assumes similar transmission conditions across communities and it is not obvious the prevalence distribution predicted by the SIS model would be observed with heterogeneous communities.[16,17] While a smaller study found a prevalence distribution in Ethiopian communities consistent with the SIS model, there is no reason to believe the findings would apply to this far larger Tanzanian survey.[9] One explanation may be that if systems tend towards states of maximum entropy over time, an exponential distribution would not be unexpected; it has the maximum entropy amongst all continuous distributions with finite mean and non-negative values.[18–20] Furthermore, infection in this cross-sectional survey was a rare event. Individual factors which normally lead to heterogeneity in transmission parameters contribute less and less as outcomes become more rare.[21]

Our study has several limitations. Models imply an exponential distribution of infection prevalence when infection is disappearing, however we only had evidence that the clinical signs of trachoma were disappearing. Because the clinical signs (trachomatous inflammation of the tarsal conjunctiva) are considered lagging indicators of infection disappears, we assumed infection must have been disappearing as well.[22] It must be noted though that while the prevalence of clinical signs of trachoma is decreasing in these areas of Tanzania from the baseline survey to this 2007–2008 survey, this 2007–2008 survey was not powered to provide district-level estimates. Furthermore, we chose to fit the prevalence data to continuous as opposed to discrete distributions because communities varied in population size. Alternatively, we could have scaled discrete distributions by the mean prevalence, as done previously.[9] Instead, we assumed that reported prevalences were a sample from a binomial distribution, given a true unobserved continuous prevalence. It is possible the prevalence data came from two different exponential distributions. To explore this, we tested a mixture exponential distribution and found that it reduced to a single exponential. Our goodness-of-fit testing assumed independence between samples. To explore this, we performed a Moran’s-I calculation. Though our Moran’s I calculation suggested there was not statistically significant geographical clustering of infection prevalences, this statistic is not perfect and there may still be some clustering. Note that if the observed data were strongly autocorrelated and we had not taken this correlation into account, then our parameter estimates would have had less precision and the exponential would have been more difficult to reject. Thus our analysis was conservative.

Our findings have several implications for trachoma control programs. An exponential distribution has a relatively heavy tail compared to a Gaussian distribution and outliers are not uncommon. Therefore we expect occasional high-prevalence communities and such communities do not necessarily suggest transmission hot spots or a failure of control efforts. In fact, models predict infection will disappear from the tail of the distribution as outliers regress to the mean, even if transmission conditions remain the same.[3,23] Reports from Nepal, Tanzania, and the Gambia have noted that infection tends to disappear in high-prevalence villages in otherwise hypo-endemic areas.[24–27]

Assessing whether trachoma control programs are on-track to eliminate infection can be difficult for public health stakeholders. Large-scale longitudinal surveys of community-wide infection prevalence are costly and resource-intensive to perform. A single cross-sectional survey, on the other hand, is much more feasible. If such a survey reveals the distribution of infection prevalence is approximated by the exponential, control programs could benefit knowing disease is on its way to elimination if transmission conditions remain the same. Further studies are needed to determine whether these findings also apply to clinical activity, the current surrogate for infection used by trachoma programs.
