# Correction: The Distribution of Ocular *Chlamydia* Prevalence across Tanzanian Communities Where Trachoma Is Declining

**DOI:** 10.1371/journal.pntd.0003783

**Published:** 2015-05-11

**Authors:** Salman A. Rahman, Sheila K. West, Harran Mkocha, Beatriz Munoz, Travis C. Porco, Jeremy D. Keenan, Thomas M. Lietman

The Funding section is incorrect. The correct funding information is as follows: The work of TCP was supported by the US National Institutes of Health, National Institute of General Medical Science, Models of Infectious Disease Agent Study (MIDAS) 1-U01GM087728 (http://www.ncbi.nlm.nih.gov/nuccore/GM087728) and the Partnership for the Rapid Elimination of Trachoma study (Bill and Melinda Gates Foundation, Grant 48027, S. West, Johns Hopkins, PI). The funders had no role in study design, data collection and analysis, decision to publish, or preparation of the manuscript.
